# 20(s)-ginseonside-Rg3 modulation of AMPK/FoxO3 signaling to attenuate mitochondrial dysfunction in a dexamethasone-injured C2C12 myotube-based model of skeletal atrophy *in vitro*

**DOI:** 10.3892/mmr.2021.12259

**Published:** 2021-06-30

**Authors:** Manying Wang, Rui Jiang, Jianzeng Liu, Xiaohao Xu, Guang Sun, Daqing Zhao, Liwei Sun

Mol Med Rep 23: Article no. 306, 2021; DOI: 10.3892/mmr.2021.11945

Following the publication of this paper, the authors requested that Daqing Zhao also be included as a joint author for correspondence. The Editor has granted this request, and therefore, the revised information for the corresponding authors is presented as follows (changes highlighted in bold):

MANYING WANG^1,2*^, RUI JIANG^1*^, JIANZENG LIU^2^, XIAOHAO XU^1^, GUANG SUN^1^, DAQING ZHAO^2,3^ and LIWEI SUWN^1,3^

^1^Research Center of Traditional Chinese Medicine, The Affiliated Hospital to Changchun University of Chinese Medicine, Changchun, Jilin 130021; ^2^Jilin Ginseng Academy, Changchun University of Chinese Medicine; ^3^Key Laboratory of Active Substances and Biological Mechanisms of Ginseng Efficacy, Ministry of Education, Changchun, Jilin 130117, P.R. China

*Correspondence to*: Professor Liwei Sun. Research Center of Traditional Chinese Medicine, The Affiliated Hospital to Changchun University of Chinese Medicine, 1478 Gongnong Street, Changchun, Jilin 130021, P.R. China

E-mail: sunnylilwei@163.com

**Professor Daqing Zhao, Jilin Ginseng Academy, Changchun University of Chinese Medicine, 1035** **Boshuo Road, Changchun, Jilin 130117, P. R. China**

**E-mail: zhaodaqing1963@163.com**

All the authors agree to this Corrigendum, and they are grateful to the Editor for allowing this Corrigendum to be published.

